# Is It Possible to Maintain Consciousness and Spontaneous Ventilation with Chest Compression in the Early Phase of Cardiac Arrest?

**DOI:** 10.1155/2016/3158015

**Published:** 2016-02-11

**Authors:** Menekse Oksar, Selim Turhanoglu

**Affiliations:** Department of Anesthesiology and Reanimation, Mustafa Kemal University Faculty of Medicine, 31100 Hatay, Turkey

## Abstract

Chest compression is important in cardiopulmonary resuscitation. However, life support algorithms do not specify when chest compression should be initiated in patients with persistent spontaneous normal breathing in the early phase after cardiac arrest. Here we describe the case of a 69-year-old man who underwent femoral bypass surgery and was extubated at the end of the procedure. After extubation, the patient's breathing pattern and respiratory rate were normal. The patient subsequently developed ventricular fibrillation, evident on two monitors. Because defibrillation was ineffective, chest compression was initiated even though the patient had spontaneous normal breathing and defensive motor reflexes, which were continued throughout resuscitation. He regained consciousness and underwent tracheal extubation without neurological sequelae on postoperative day 1. This case highlights the necessity of chest compression in the early phase of cardiac arrest.

## 1. Introduction

The 2010 American Heart Association recommendations and European Resuscitation Council guidelines for cardiopulmonary resuscitation (CPR) focus on the requirement of immediately initiating chest compression and ventilation to maintain cerebral blood flow and adequate gas exchange, respectively [1]. Maintaining cerebral perfusion prevents neurological damage. Some studies have shown that conscious, spontaneous breathing may continue for a short time after cerebral perfusion stops; for example, repeated rhythmic coughs every 1–3 s maintained consciousness for up to 39 s in three patients who developed ventricular fibrillation during coronary arteriography [2]. Cardiac arrest survivors recall memories of awareness, fear, and persecution after CPR [3]. Early diagnosis of cardiac arrest may prolong spontaneous breathing and consciousness by maintaining cerebral perfusion through effective chest compression. We present a 69-year-old man with uninterrupted spontaneous normal breathing during CPR.

## 2. Case Report

A 69-year-old man (American Society of Anesthesiologists class 2; weight 70 kg; height 1.72 m) with peripheral arterial disease and diabetes mellitus underwent femoral bypass surgery. Anesthesia was induced using 2 mg midazolam, 100 *μ*g fentanyl, and 2 mg/kg propofol. Endotracheal intubation was achieved with 40 mg rocuronium, anesthesia was maintained by 2% sevoflurane with N_2_O and 50% O_2_, and diuretic and insulin infusions were administered as required. The patient showed normal blood gas levels (pH, 7.36; PaCO_2_, 43 mmHg; PaO_2_, 92 mmHg; lactate, 3.5 mmol/L; and base excess, 1.2 mmol/L). His blood glucose level was 220 mg/dL, O_2_ saturation level was 99% on 50% O_2_, heart rate (HR) was 88 beats/min, and mean arterial pressure (MAP) was 70 mmHg. Neuromuscular blockade was reversed using 200 mg sugammadex, and the patient was extubated. Thereafter, his breathing pattern was regular, with a respiratory rate of 17 breaths/min and a tidal volume of 600 mL. Oxyhemoglobin saturation determined using pulse oximetry was 99%, invasive arterial blood pressure was 140/90 mmHg (MAP, 106 mmHg), and HR was 100 beats/min. Further, a transport monitor was connected.

The patient subsequently developed ventricular fibrillation (VF), evident on 2 monitors. A biphasic 150-J shock was administered immediately, and ventilation was initiated with a facemask supplying O_2_ at 6 L/min before his trachea was reintubated without a neuromuscular blocking agent. Ventilatory support was manually administered because he was breathing spontaneously. After the first shock, external cardiac massage was applied for 2 min. However, the patient remained in VF; therefore, a second shock was administered, followed by further chest compression. The patient became asystolic and received 1 mg intravenous epinephrine. Although spontaneous breathing persisted, CPR was continued. Shocks and chest compression continued for 1 h, with epinephrine administered every 3–5 min with short breaks to assess cardiac rhythm. The chest compression evoked defensive motor reflexes, such as flexion of the neck, trunk, and arms, and arterial traces were determined to originate from external cardiac massage during resuscitation.

During resuscitation, arterial blood gas recordings showed acidosis with an abnormally low pH, high lactate, and low base excess. PaCO_2_ was normal during VF and resuscitation, but it increased by the end of resuscitation. Although assisted breathing was provided throughout resuscitation, the rate of spontaneous breathing was 16-17 breaths/min with a tidal volume of 600 mL/min. During resuscitation, end-tidal CO_2_ was 19–22 mmHg. The potassium level was normal intraoperatively (3.6 mmol/L), reduced during resuscitation (2.0 mmol/L), and increased with potassium replacement therapy by the end of resuscitation (3.2 mmol/L). The patient remained asystolic until circulation resumed spontaneously. After resuscitation, the HR was recorded at 51 beats/min with ST depression observed on electrocardiography (ECG) (Table 1). Measurements recorded at baseline, preoperatively, during CPR, and after CPR are shown in Table 1. Spontaneous breathing continued without interruption throughout the entire CPR period. Following tracheal intubation, ventilation was manually assisted with the balloon of a breathing circuit throughout the entire CPR period and an Ambu bag during patient transfer following resuscitation. The actual measured duration of asystole was 50 min. VF lasted for approximately 10 minutes. During cardiac arrest, defibrillation attempts were made and chest compression was initiated in order to maintain adequate blood pressure. CPR was continued until potential underlying correctable causes of VF (such as the hypokalemia and low arterial pH levels in the present case) were determined and corrective interventions could be performed. Approximately 1 hour after correction of the metabolic causes of VF, during which spontaneous breathing and continued motor responses to chest compression without QRS complexes on electrocardiography were observed, the patient was reviewed by a cardiologist and was subsequently transferred to coronary angiography for further investigations. ECG and ventilatory data at the end of CPR were given as supplementary material (Figure S1 in Supplementary Material available online at http://dx.doi.org/10.1155/2016/3158015).

During angiography, the patient again developed asystole requiring further chest compression but responded to CPR and was transferred to the cardiovascular surgery intensive care unit. Coronary angiography was reported as normal. The patient regained consciousness on postoperative day 1, was extubated, and showed no evidence of neurological sequelae. Written consent for the publication of this report was obtained from the patient postoperatively.

## 3. Discussion

A significantly increased incidence of ventricular tachycardia (VT) and VF has been reported after reperfusion therapy for patients with myocardial infarction and systemic acidosis [4]. There is evidence that systemic metabolic acidosis is a strong predictor of VT/VF after reperfusion of ST-elevation myocardial infarction associated with inflammation [5]. However, the causal relationship between systemic acidosis secondary to myocardial ischemia and VT/VF is complex, and the trigger is not always obvious. In this case, the lactic acidosis after surgical clamping coupled with the final low HCO_3_^−^ may have created a high-anion-gap metabolic acidosis that caused VF. Another possibility is that using insulin or diuretics caused hypokalemia, which induced VF.

Although VF is the most frequent cause of cardiac arrest and death, it is reversible if treated early. Failure to commence CPR between cardiac arrest and the cessation of spontaneous breathing may cause irreversible brain injury. Therefore, current guidelines recommend immediate chest compression for unresponsive patients who are breathing abnormally [6]. For spontaneously breathing patients who are unresponsive or unconscious, guidelines recommended they should be placed in the recovery position because spontaneous breathing is considered a reliable sign of effective cardiac activity [1]. However, a few reports have demonstrated that normal spontaneous breathing, and even consciousness, continues for a short time after a cardiac arrest.

In one study of 8 patients, breathing pattern, frequency, and tidal volume were found to be unchanged in the first 12–15 s of VF [7]. These findings were confirmed in adult sheep, where breathing patterns were unchanged for 15 s and minute ventilation increased for 55 ± 44 s before stopping abruptly. Agonal breathing efforts have been reported to begin 37 ± 38 s into the apneic period, whereas there is evidence that normal breathing ceases after 12–60 s and that agonal breathing can persist for up to 6 min [8]. Agonal breathing was reported in 40% of 445 out-of-hospital cardiac arrests, where it was more frequent in patients with VF and was associated with increased survival [9]. Our patient continued to breathe spontaneously throughout CPR, perhaps because of the early and uninterrupted breathing support, and we did not detect agonal breathing.

As mentioned previously, rhythmic coughs have been shown to prolong consciousness in VF [2], whereas survivors of cardiac arrest have reported a broad range of cognitive experiences. Furthermore, 2% (2 of 101 patients) have reported retaining full awareness [3]. Although it is unclear whether the experiences during cardiac arrest refer to actual events or hallucinations, the finding that conscious awareness can remain during cardiac arrest is intriguing and supports other recent studies indicating that consciousness may be present despite clinically undetectable consciousness. Another report has suggested that functional neuroimaging techniques can assist with the prognosis and diagnosis of patients with disorders of consciousness [10]. Notably, our patient displayed defensive motor reflexes in response to painful stimuli (chest compression) but denied any recollection.

In conclusion, because respiratory neurons can maintain respiration immediately after cardiac arrest, it can be misleading to assume that any normal breathing movements preclude a diagnosis of cardiac arrest. Therefore, the first 10–15 s of a witnessed cardiac arrest are crucial to save patients' lives and prevent neurological sequelae. Although intervention during this time is critical, it will be difficult to implement appropriate interventions with unmonitored patients.

## Supplementary Material

Figure S1: Screenshots of the electrocardiography (ECG) and ventilator monitors at the end of the resuscitation. The patient consulted with a cardiologist and provided mechanical ventilation during preparation for discharge from the operating room. The ECG monitor displayed the heart rate. However, regular QRS complexes were not recognized. According to the cardiologist, the printed ECG did not show QRS complexes at that time. Traces observed on the anesthesia machine ventilator screen from the top to the bottom are as follows: ETCO_2_, tidal volume (it is also denoted as mL), minute ventilation, breathing frequency, peak pressure, plateau pressure, and PEEP pressure. The ETCO_2_ level did not differ from the pre-arrest levels over the entire resuscitation period.

## Figures and Tables

**Table 1:** Perioperative arterial blood gas, peak end-tidal partial CO_2_ pressure, and concomitant hemodynamic parameter measurements (*bold column*, *time of VF initiation*; *italic columns*, *cardiac arrest period*).

Measurement number	T1	T2	**T3**	*T4*	*T5*	*T6*	*T7*	T8	T9	T10
pH	7.45	7.36	**7.01**	*No result*	*7.19*	*7.26*	*7.05*	7.12	7.39	7.40
PaCO_2_ (mmHg)	40	43	**39**	*No result*	*47*	*61*	*78*	56	34	68
ETCO_2_ (mmHg)	25	25	**Not monitored ** **ETCO**_**2**_	*Not monitored ETCO*_*2*_	*19*	*19*	*21*	22	Not monitored	Not monitored
Base excess	3.5	−1.2	**−21**	*No result*	*−9.9*	*−0.2*	*−9.3*	−10.7	−3.7	−3.1
PaO_2_ (mmHg)			**126**	*No result*				92	93	68
Lactate (mMol/L)	2.0	3.5	**7.5**	*7.5*	*8.0*	*8.0*	*8.5*	11.7	6.0	3.5
Glucose (mg/dL)	126	220	**205**					256		
Potassium (mMol/L)	3.5	3.6	**2.0**	*2.2*	*2.8*	*3.0*	*3.2*	4.0	3.6	3.2
FiO_2_ (%)	50	50	**Room air**	*100*	*100*	*100*	*100*	100	100	Room air
Cardiac rhythm	Sinus	Sinus	**VF**	*Asystole*	*Asystole*	*Asystole*	*Asystole*	Bradycardia	Sinus	Sinus
HR (beat/minute)	67	70	**Not applicable**	*0*	*100*	*100*	*100*	51	80	88
MAP (mmHg)	60	60	** No pressure**	*No pressure*	*60*	*65*	*65*	60	68	85
SpO_2_ (%)	99	99	**Unreadable**	*Unreadable*	*Unreadable*	*Unreadable*	*Unreadable*	Unreadable	97	95

CPR, cardiopulmonary resuscitation; ETCO_2_, peak end-tidal partial pressure of CO_2_; FiO_2_, fractional inspired O_2_ concentration; HR, heart rate; MAP, mean arterial pressure; PaCO_2_, arterial partial pressure of CO_2_; PaO_2_, arterial partial pressure of O_2_; SpO_2_, O_2_ saturation; VF, ventricular fibrillation.

T1, baseline; T2, intraoperative period; T3, postoperative VF and defibrillation; T4, after 2 min of CPR after defibrillation attempts; T5, resuscitation 1; T6, resuscitation 2; T7, resuscitation 3; T8, resuscitation 4; T9, postoperative day 1; T10, after tracheal extubation on postoperative day 1.
