# Supplementary figures and images for: Is It Possible to Maintain Consciousness and Spontaneous Ventilation with Chest Compression in the Early Phase of Cardiac Arrest?
Source: Case Rep Anesthesiol. 2016 Feb 11;2016:3158015. doi: 10.1155/2016/3158015 (PMC4766314; doi:10.1155/2016/3158015)

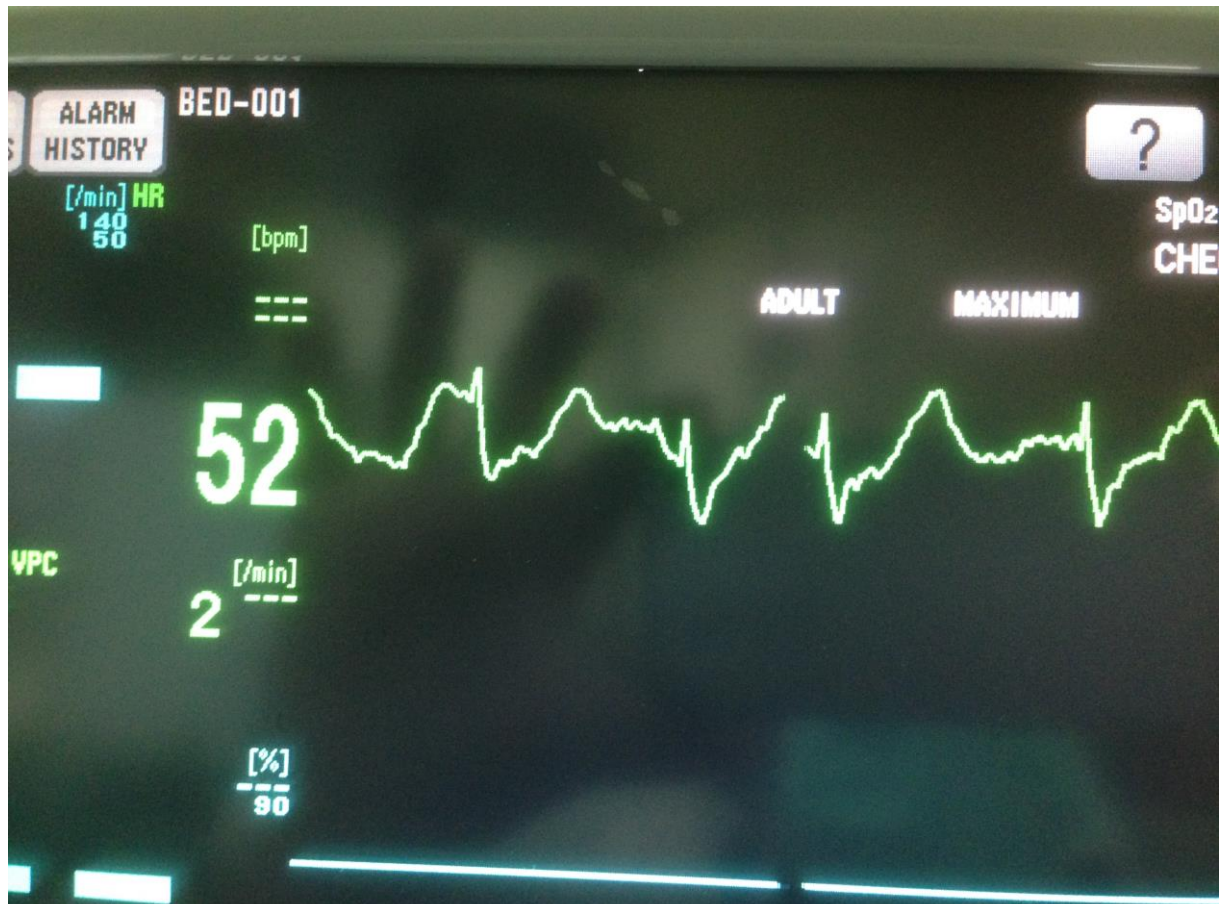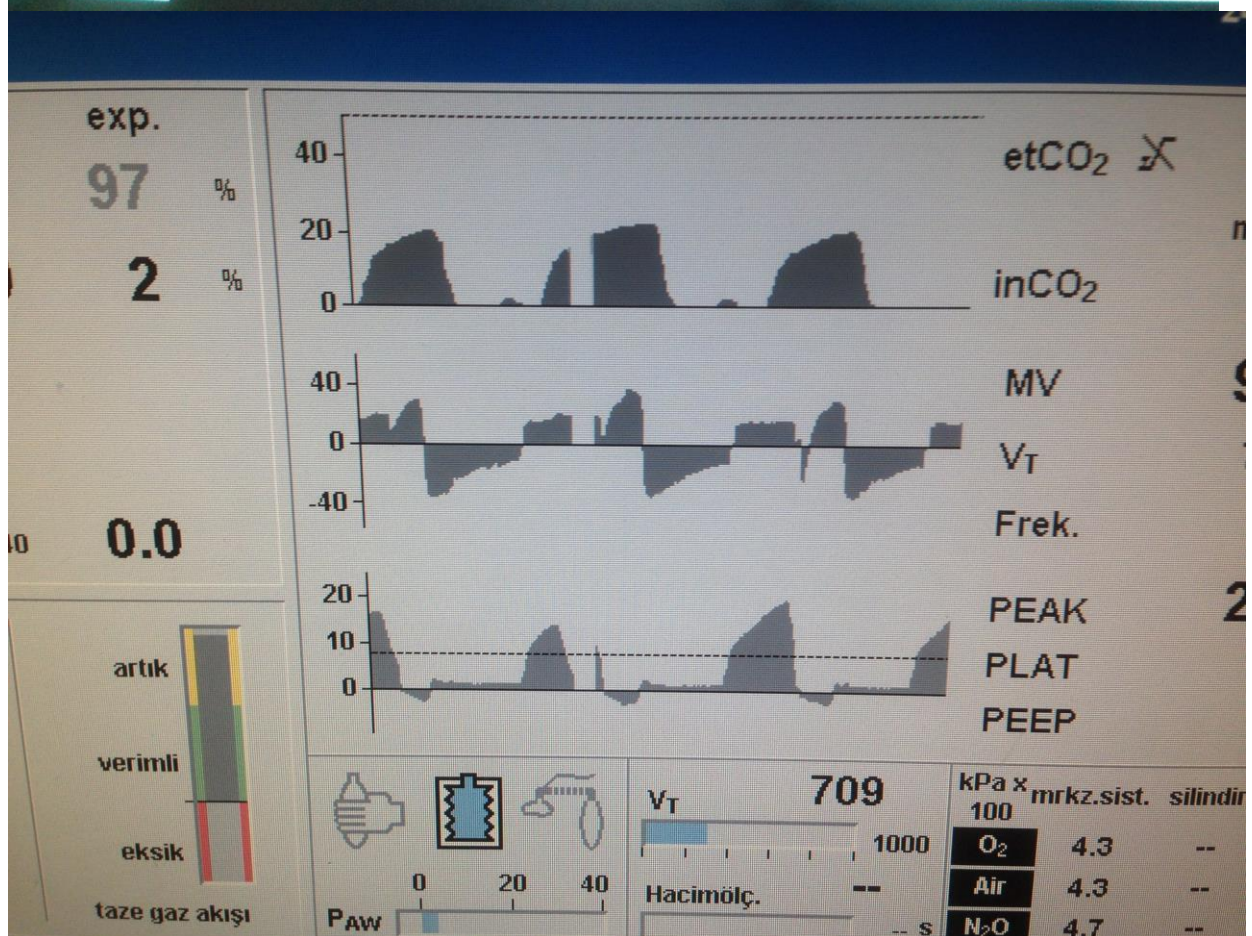

Supplement: Supplementary file 1 — Figure S1: Screenshots of the electrocardiography (ECG) and ventilator monitors at the end of the resuscitation. The patient consulted with a cardiologist and provided mechanical ventilation during preparation for discharge from the operating room. The ECG monitor displayed the heart rate. However, regular QRS complexes were not recognized. According to the cardiologist, the printed ECG did not show QRS complexes at that time. Traces observed on the anesthesia machine ventilator screen from the top to the bottom are as follows: ETCO2, tidal volume (it is also denoted as mL), minute ventilation, breathing frequency, peak pressure, plateau pressure, and PEEP pressure. The ETCO2 level did not differ from the pre-arrest levels over the entire resuscitation period. [file 3158015.f1.pdf]
